# Myeloid-derived suppressor cells inhibit T cell proliferation in human extranodal NK/T cell lymphoma: a novel prognostic indicator

**DOI:** 10.1007/s00262-015-1765-6

**Published:** 2015-10-23

**Authors:** Han Zhang, Ze-Lei Li, Shu-Biao Ye, Li-Ying Ouyang, Yu-Shan Chen, Jia He, Hui-Qiang Huang, Yi-Xin Zeng, Xiao-Shi Zhang, Jiang Li

**Affiliations:** 1 State Key Laboratory of Oncology in South China, Sun Yat-Sen University Cancer Center, 651 Dongfeng East Road, Guangzhou, 510060 China; 2 Collaborative Innovation Center of Cancer Medicine, Sun Yat-Sen University Cancer Center, 651 Dongfeng East Road, Guangzhou, 510060 China; 3 Department of Biotherapy, Sun Yat-Sen University Cancer Center, 651 Dongfeng East Road, Guangzhou, 510060 China; 4 Department of Medical Oncology, Sun Yat-Sen University Cancer Center, 651 Dongfeng East Road, Guangzhou, 510060 China; 5 Intensive Care Unit Department, Sun Yat-Sen University Cancer Center, 651 Dongfeng East Road, Guangzhou, 510060 China; 6 Department of Radiotherapy, Sun Yat-Sen University Cancer Center, 651 Dongfeng East Road, Guangzhou, 510060 China

**Keywords:** Prognosis, Immunosuppression, Hematopoietic malignancy, MDSCs, NK/T cell lymphoma

## Abstract

The expansion of myeloid-derived suppressor cells (MDSCs) and its correlation with advanced disease stage have been shown in solid cancers. Here, we investigated the functional features and clinical significance of MDSCs in extranodal NK/T cell lymphoma (ENKL). A higher percentage of circulating HLA-DR^−^CD33^+^CD11b^+^ MDSCs was observed in ENKL patients than in healthy controls (*P* < 0.05, *n* = 32) by flow cytometry analysis. These MDSCs from ENKL patients (ENKL-MDSCs) consisted of CD14^+^ monocytic (Mo-MDSCs, >60 %) and CD15^+^ granulocytic (PMN-MDSCs, <20 %) MDSCs. Furthermore, these ENKL-MDSCs expressed higher levels of Arg-1, iNOS and IL-17 compared to the levels of MDSCs from healthy donors, and they expressed moderate levels of TGFβ and IL-10 but lower levels of CD66b. The ENKL-MDSCs strongly suppressed the anti-CD3-induced allogeneic and autologous CD4 T cell proliferation (*P* < 0.05), but they only slightly suppressed CD8 T cell proliferation (*P* > 0.05). Interestingly, ENKL-MDSCs inhibited the secretion of IFNγ but promoted IL-10, IL-17 and TGFβ secretion as well as Foxp3 expression in T cells. The administration of inhibitors of iNOS, Arg-1 and ROS significantly reversed the suppression of anti-CD3-induced T cell proliferation by MDSCs (*P* < 0.05). Importantly, based on multivariate Cox regression analysis, the HLA-DR^−^CD33^+^CD11b^+^ cells and CD14^+^ Mo-MDSCs were independent predictors for disease-free survival (DFS, *P* = 0.013 and 0.016) and overall survival (OS, *P* = 0.017 and 0.027). Overall, our results identified for the first time that ENKL-MDSCs (mainly Mo-MDSCs) have a prognostic value for patients and a suppressive function on T cell proliferation.

**Electronic supplementary material:**

The online version of this article (doi:10.1007/s00262-015-1765-6) contains supplementary material, which is available to authorized users.

## Introduction

Extranodal natural killer (NK)/T cell lymphoma (ENKL) has distinct epidemiological, clinical, histological and etiological features. Clinically, ENKL predominantly occurs in the nasal–paranasal area, skin, gastrointestinal tract or other extranodal sites, and it has a poor prognosis caused by rapid lesion progression [1]. Among the Epstein–Barr virus (EBV)-related lymphomas including Hodgkin lymphoma and Burkitt lymphoma, ENKL is the one most closely associated with EBV infection. EBV latent type II antigens, including latent membrane protein-1 and protein-2 (LMP1 and LMP2) and EBV nuclear antigen 1 (EBNA1), are present in ENKL tumor cells. Immune imbalance has been shown to be an important feature of ENKL patients [2, 3]. However, the role of immune cells during ENKL progression remains largely unclear.

Myeloid-derived suppressor cells (MDSCs) are a heterogeneous population of bone marrow-derived myeloid progenitors including macrophages, granulocytes, dendritic cells and immature myeloid cells [4, 5]. Studies in recent years have revealed that MDSCs expand dramatically during tumor growth and are a cause of immune evasion of many types of tumors, including multiple myeloma [6, 7]. MDSCs enhance tumor growth by inhibiting immune responses and T cell proliferation as well as facilitating tumor metastasis and angiogenesis [8–12]. MDSCs can inhibit anti-tumor immunity by suppressing T cell and NK cell functions by increasing the production of arginine, reactive oxygen species (ROS) and nitric oxide (NO) as well as by inducing Treg cells and TGF-β secretion to mediate T cell suppression [13–15]. To our knowledge, the role of MDSCs, a novel immune-suppressive cell subset, during ENKL tumor progression has not previously been reported. In this study, we detected the frequency of MDSCs in the peripheral blood of ENKL patients to characterize the phenotypic and functional features of MDSCs in ENKL, and we further assessed its clinical significance and prognostic value.

## Materials and methods

### Patients

Peripheral blood mononuclear cells (PBMCs) were collected from 32 age-matched healthy donors and 32 patients with ENKL at the first time of diagnosis at Sun Yat-Sen University Cancer Center (Guangzhou, China) from July 2010 to December 2012. The clinical details of the patients are shown in Supplementary Table 1. All patients were diagnosed with ENKL, and the lymphoma involved nasal and paranasal lesions in 25 cases (upper aerodigestive tract NK/T cell lymphoma, UNKTL; 84.4 %). The median age was 40.5 years old, and the age range was from 17 to 70 years. There were 19 patients in stage I, 3 patients in stage II, 3 patients in stage III and 7 patients in stage IV. Nine patients had elevated serum lactate dehydrogenase (LDH) levels, and 20 patients had B symptoms. The International Prognostic Index (IPI) was high-intermediate/high (2–5) in eight patients. For the Korean Prognostic Index (KPI) model, 17 patients (53.1 %) had none or one adverse factor, and 15 patients (46.9 %) had two to four adverse factors. In the Peripheral T cell lymphoma Prognostic Index (PIT) model, the majority of the patients (20 cases, 62.5 %) had none or one adverse factor, and the other 12 cases (37.5 %) had at least two adverse factors. Nine of the 32 patients were deceased, and the 5-year overall survival was 71.9 % with a median follow-up of 52 months.

All patients and healthy donors provided informed consent prior to the blood sampling. The study was approved by the Research Ethics Committee of the Sun Yat-Sen University Cancer Center.

### Flow cytometry analysis

Human monoclonal antibodies against HLA-DR, CD33, CD11b, CD14, CD15, CD66b, iNOS, Arg-1, IL-10, IL-17 and TGFβ conjugated to different fluorescent dyes were obtained from BD Pharmingen (San Jose, CA, USA) or eBioscience (San Diego, CA, USA), and they were used to measure the frequency and phenotype of the MDSCs via surface staining or intracellular staining (Supplementary Table 2). PBMCs were isolated via Ficoll-Hypaque gradient centrifugation to measure the proportion and phenotype of MDSCs. For surface staining, the cells were washed twice and stained for 1 h on ice with mixtures of fluorescence-conjugated surface mAbs or isotype-matched controls. The cells were then washed twice and resuspended in PBS buffer for flow cytometry analysis. The intracellular staining of IL-17 and the other cytokines was performed on PBMCs stimulated with lipopolysaccharide (LPS, 1 μg/ml) for 4 h in RPMI 1640 medium, and the cytokine secretion was blocked by the addition of brefeldin A (10 µg/ml, eBioscience). After washing, the cells were stained with anti-CD33, anti-CD11b and anti-HLA-DR. The cells were then fixed, permeabilized with Perm/Fix solution (eBiosciences) and stained intracellularly with anti-IL-17 or fluorescence-conjugated antibodies for other cytokines. The samples were evaluated on a FC500 flow cytometer (Beckman Coulter) and analyzed with CXP Software (Beckman Coulter, Inc., Fullerton, CA, USA).

### T cell suppression assay

CD33^+^ cells were isolated from the PBMCs from the healthy donors or ENKL patients using human CD33 MicroBeads (Miltenyi Biotec, Bergisch Gladbach, Germany) according to the manufacturer’s instructions. The PBMCs from healthy donors were labeled with 5 μM carboxyfluorescein succinimidyl ester (CFSE; Molecular Probes, Eugene, Oregon, USA) in 1 ml of PBS for 15 min at 37 °C. The labeling was halted by adding an excess of FCS, and the samples were washed twice with RPMI 1640 (Gibco, Life Technologies, China) supplemented with 10 % fetal bovine serum (FBS; ExCell Biology, South America). The CSFE-labeled cells were cultured in an anti-CD3 antibody (OKT3)-coated 96-well plate with or without sorting the CD33^+^ cells from the ENKL patients or healthy donors at different ratios for 3 days, and *N*-hydroxy-nor-l-arginine (NOHA; 1 mM), l-NG-monomethylarginine (l-NMMA, 100 μM) or *N*-acetylcysteine (NAC 1, mM) was added to a portion of the samples. The CFSE fluorescence intensity was analyzed by flow cytometry after 7 days of co-culture and proliferation.

### Statistical analyses

The numerical data are shown as the mean ± standard error (SEM). The statistical analysis was performed with the SPSS 13.0 software (SPSS, Chicago, IL, USA) or GraphPad Prism analysis tools (La Jolla, CA, USA). Two group comparisons were tested using Student’s *t* test, and the association of the density of the MDSCs with the clinical pathological features was examined using Pearson’s chi-square test. The overall survival (OS) was measured from the date of the diagnosis to the date of death from any cause or to the date of the last follow-up visit. The disease-free survival (DFS) was defined as the time from the diagnosis to the first occurrence of progression, relapse after a response, death from any cause, or to the date of the last follow-up of the surviving patients. The survival curves were determined by the Kaplan–Meier method and the log-rank test. A Cox proportional hazards regression analysis was performed to identify the independent prognostic factors for the OS or DFS. The cutoff value was the median of all variants. The statistical tests were based on a level of significance at *P* < 0.05.

## Results

### The expansion and clinical implication of circulating MDSCs in ENKL

**Fig. 1:**
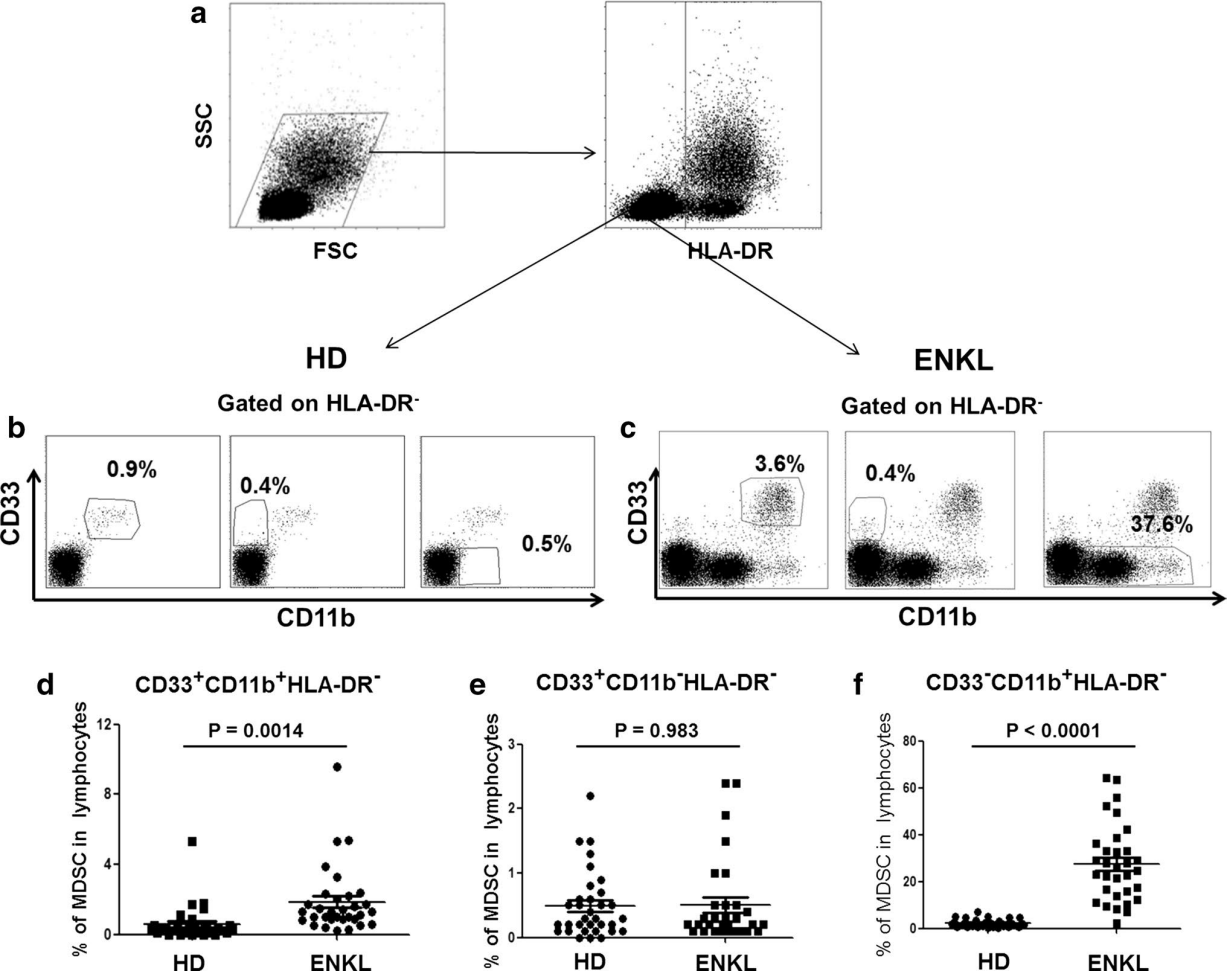
Expansion of MDSCs in patients with extranodal NK/T cell lymphoma. **a** Gating routine for MDSC subsets. **b**–**c** The *dot plots* represent the CD33^+^CD11b^+^ cell subset, the CD33^+^CD11b^−^ cell subset and the CD33^−^CD11b^+^ cell subset gating on the HLA-DR^−^ fraction among the PBMCs from healthy donors (**b**) or ENKL patients (**c**). **d**–**f** The statistical analysis of the percentage of the MDSC subsets among the PBMCs from the ENKL patients (*n* = 32) and healthy donors (*n* = 32). The *error bar* represents the SEM. Student’s *t* test is used. *HD* healthy donor, *ENKL* extranodal NK/T cell lymphoma

We investigated the frequency of MDSCs in the peripheral blood mononuclear cells (PBMCs) of 32 ENKL patients. Flow cytometry analysis showed that the percentage of HLA-DR^−^CD33^+^CD11b^+^ and HLA-DR^−^CD33^−^CD11b^+^ cells was increased in the PBMCs from ENKL patients compared with those from healthy controls (*P* = 0.0014 and *P* = 0.0001, respectively) as shown in Fig. 1. No correlation between the frequency of MDSC populations and clinicopathological factors, including age, gender, Ann Arbor Stage, subtypes, LDH level, B symptoms, KPI, PIT and IPI scores, was observed (*P* > 0.05) as shown in Supplementary Table 3. Further, no correlation was found between the frequency of the circulating CD14^+^ monocytic (Mo-MDSCs) or CD15^+^ granulocytic (PMN-MDSCs) subsets and clinicopathological parameters (*P* > 0.05, Supplementary Table 4).

### The phenotypic properties and cytokine profile of MDSCs in ENKL

**Fig. 2:**
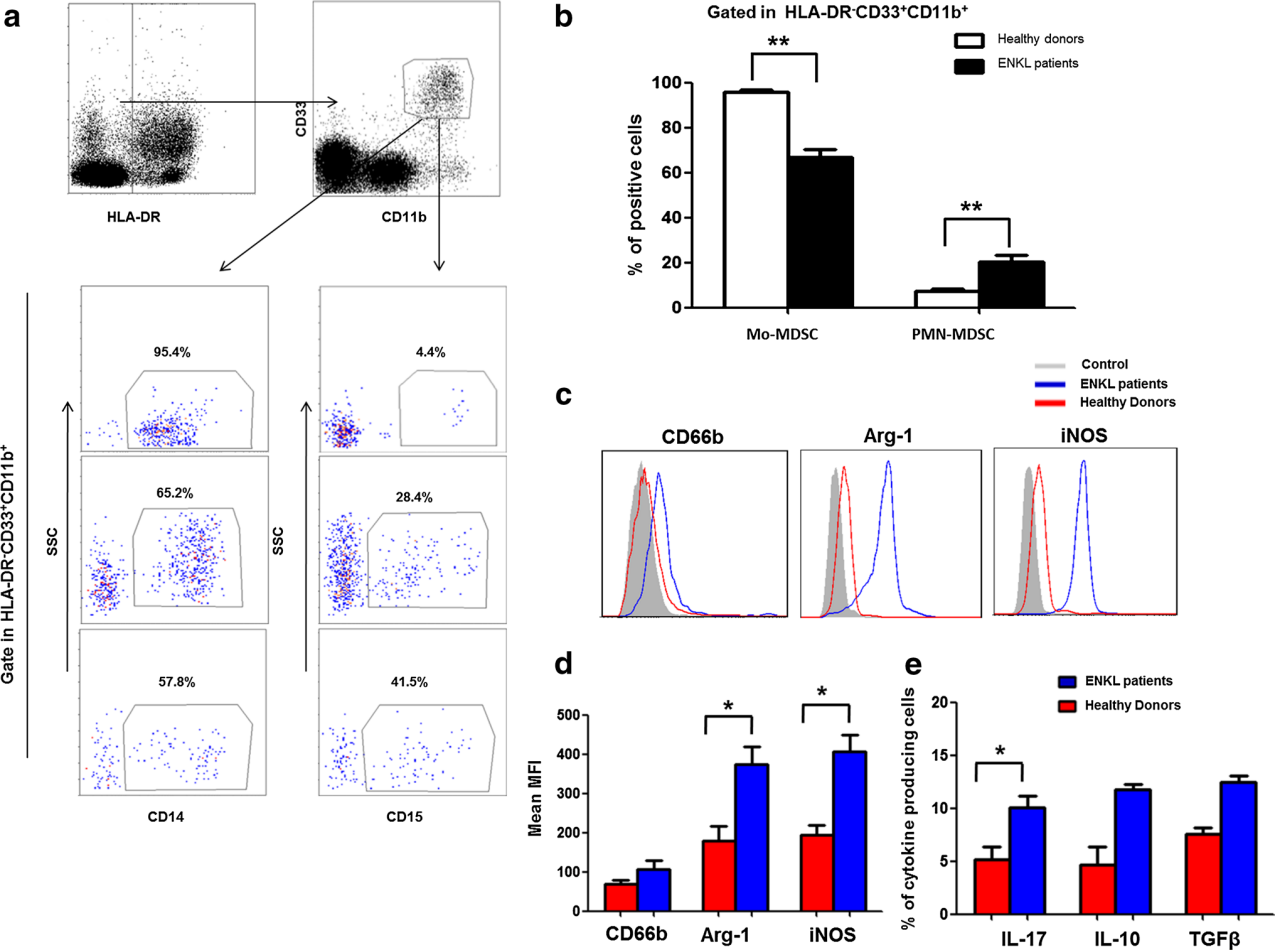
Phenotypes and cytokine profiles of HLA-DR^−^CD33^+^CD11b^+^ MDSCs in extranodal NK/T cell lymphoma patients. The HLA-DR^−^CD33^+^CD11b^+^ cells are gated as MDSCs from 22 NK/T cell lymphoma patients. The properties of the MDSCs are analyzed via flow cytometry using multiple antihuman mAbs against CD14, CD15, CD66b, iNOS, Arg-1, IL-17, IL-10 and TGFβ. **a** Representative FACS plots of the CD14^+^ or CD15^+^ MDSCs from the same ENKL patients. **b** Graph of the CD14^+^ Mo-MDSCs and CD15^+^ PNM-MDSCs among the PBMCs from 22 ENKL patients and 22 healthy controls. **c** Representative FACS histogram for CD66b, iNOS and Arg-1 expression in ENKL-MDSCs and MDSCs from healthy control. **d** The data shown are the MFI of CD66b, iNOS and Arg-1 in ENKL-MDSCs from 22 ENKL patients and MDSCs from healthy controls determined by cytofluorimetric analysis and are corrected for background staining. **e** The percentage of cytokine-producing ENKL-MDSCs from 22 ENKL patients and MDSCs from healthy controls, including IL-17, IL-10 and TGFβ. MFI, mean fluorescence intensity; **P < 0.01; *P < 0.05

Based on a previous study [12], we described the HLA-DR^−^CD33^+^CD11b^+^ cells as MDSCs in subsequent experiments. To evaluate the phenotypic properties of this population in the PBMCs from patients with ENKL (ENKL-MDSCs), we screened the specific markers and cytokines of ENKL-MDSCs using FACS analysis and a multiple gate strategy. First, based on previous reports, we determined that the ENKL-MDSCs predominantly consisted of CD14^+^ Mo-MDSCs (>60 %), and the CD15^+^ PMN-MDSC subset represented approximately 20 % of the MDSC population of ENKL patients. The proportion of Mo-MDSCs and PMN-MDSCs in the peripheral blood of ENKL patients was significantly different to that of healthy donors (*P* < 0.05, Fig. 2a, b). Furthermore, the ENKL-MDSCs displayed a significantly higher level of Arg-1 and iNOS compared to healthy donors (*P* < 0.05), and the MDSCs from both ENKL patients and healthy donors displayed a moderate level of CD66b as shown in Fig. 2c, d. In addition, we found that the MDSCs secreted a moderate level of IL-17, IL-10 and TGFβ. Interestingly, the ENKL-MDSCs secreted a significantly higher level of IL-17 (*P* < 0.05) and a slightly higher level of IL-10 and TGFβ compared to the levels of the MDSCs from healthy donors (Fig. 2e).

### ENKL-MDSC-mediated suppression of T cell proliferation is dependent on NO and ROS production

**Fig. 3:**
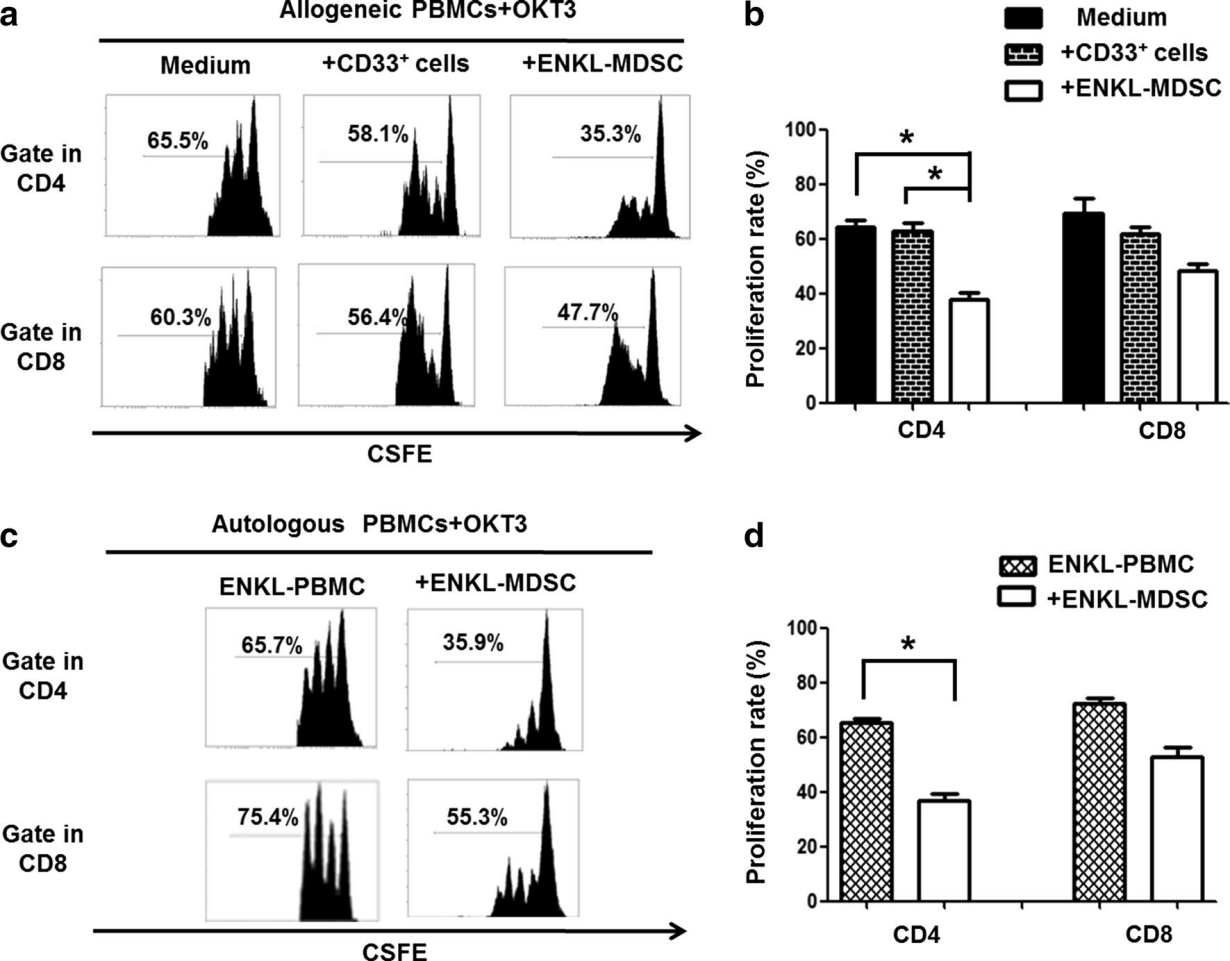
ENKL-MDSCs suppress allogeneic and autologous T cell proliferation. T cell proliferation is examined by CSFE labeling in vitro. The CD33^+^ cells are sorted from the PBMCs from five patients with ENKL, and CD33^+^ cells from healthy donors are included as a control. The CSFE-labeled PBMCs are co-cultured with the CD33^+^ cells at a ratio of 2:1 in OKT3-coated 96-well plates. After 3 days, the cells are collected and quantified using flow cytometry. **a**, **c** Allogeneic and autologous OKT3-stimulated PBMCs. Representative FACS density plots from one of the five experiments. **b**, **d** The graph of the statistical analyses is presented. The *error bars* represent the SEM. *n* = 5; *P < 0.05; *HD* healthy donors

To further understand the role of MDSCs in ENKL progression, we investigated the immunosuppressive function of MDSCs isolated from the PBMCs of ENKL patients. The CD33^+^ cells isolated from the ENKL patients showed noticeable inhibition of the proliferation of allogeneic and autologous OKT3-stimulated CD4 T cells (*P* < 0.05), but only a slight suppression of allogeneic and autologous OKT3-stimulated CD8 T cell proliferation was observed (*P* > 0.05) as shown in Fig. 3. Our observations indicated that the ENKL-MDSCs displayed a suppressive function dependent on MHC limitation and non-specific suppression, especially for CD4 T cell proliferation.

**Fig. 4:**
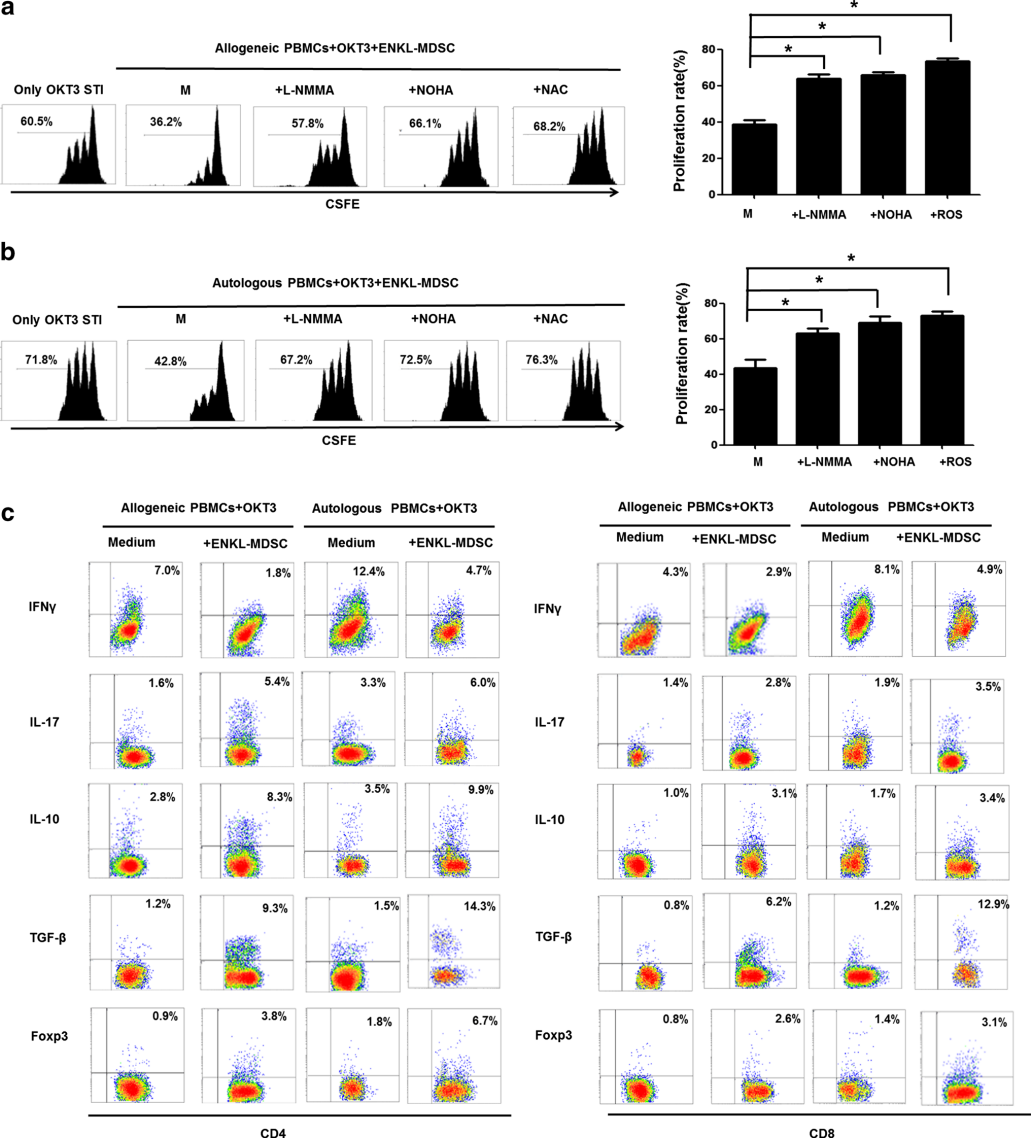
Multiplex mechanisms are involved in the ENKL-MDSC-mediated suppression of T cell proliferation. l-NMMA, NOHA or NAC is added to a portion of the samples in the co-culture system of CSFE-labeled PBMCs and ENKL-MDSCs at ratio 2:1 in OKT3-coated 96-well plates. After 3 days, the cells are collected and quantified using flow cytometry. **a**, **b** Allogeneic and autologous OKT3-stimulated PBMCs. **c** Cytokine secretion (IFNγ, IL-17, IL-10 and TGFβ) and Foxp3 expression in CD4 or CD8 T cells in the presence of allogeneic ENKL-MDSCs, autologous ENKL-MDSCs or only in medium. l-NMMA, NG-methyl-l-arginine; NOHA, *N*-hydroxy-nor-l-arginine; NAC, *N*-acetylcysteine; Student’s *t* test is used

Subsequently, we further explored the potential suppressive mechanism of MDSCs in ENKL. Firstly, in the ENKL-MDSC population, iNOS and Arg-1 were highly expressed (Fig. 2c, d). iNOS and Arg-1 are key enzymes responsible for arginine metabolism and the production of NO, respectively. These enzymes share the same substrate, l-arginine, and are associated with MDSC-mediated suppression [16, 17]. We further investigated the underlying mechanisms controlling MDSC-mediated T cell suppression in ENKL by blocking the activity of iNOS, Arg-1 and ROS production in MDSCs. Suppression of T cells mediated by CD33^+^ cells isolated from the ENKL patients was almost completely recovered after administration of the arginase inhibitor (NOHA), the nitric oxide synthase inhibitor (l-NMMA) or the ROS inhibitor (NAC) (Fig. 4a, b). When OKT3-stimulated CD4 or CD8 T cells were co-cultured with MDSCs from ENKL patients for 3 days, we found that the secretion of IL-10, TGFβ and IL-17 as well as Foxp3 expression were significantly increased, while the secretion of IFNγ was significantly decreased (Fig. 4c). These data suggested that when T cells are co-cultured with ENKL-MDSCs, the altered cytokine secretion from T cells, including increased IL-10 and TGFβ secretion, as well as induction of Foxp3^+^ Treg cells, suppresses T cell proliferation.

### The correlation of MDSC populations and ENKL patient prognosis

**Fig. 5:**
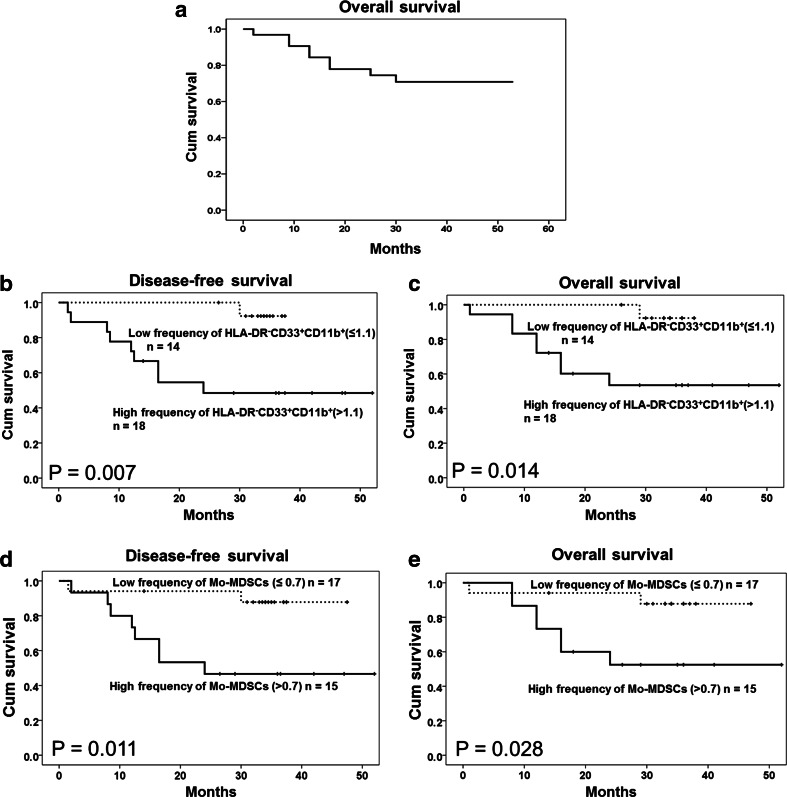
Correlation of circulating MDSCs or Mo-MDSCs with survival in extranodal NK/T cell lymphoma cases. **a** The overall survival (OS) curve of 32 ENKL patients. **b**, **c** The DFS and OS rates are significantly different between the high and low HLA-DR^−^CD33^+^CD11b^+^ cell counts (*P* = 0.007 and 0.014, respectively, log-rank test). **d**, **e** The DFS and OS rates are significantly different between the high and low CD14^+^ Mo-MDSC counts (*P* = 0.011 and 0.028, respectively, log-rank test). The cutoff value is the median of the HLA-DR^−^CD33^+^CD11b^+^ cell or CD14^+^ Mo-MDSC density

Nine patients (28.1 %) had died by the time of analysis (Supplementary Table 1), and the patients with a higher frequency of circulating HLA-DR^−^CD33^+^CD11b^+^ MDSCs and CD14^+^ Mo-MDSCs, which are the main composition of MDSC populations, had shorter DFS (*P* = 0.007 and 0.011) and OS (*P* = 0.014 and 0.028) (Fig. 5). However, no association was found between patient survival and the frequency of CD15^+^ PMN-MDSCs or HLA-DR^−^CD33^−^CD11b^+^ cells in ENKL patients (*P* > 0.05, Supplementary Figure 1). In addition to the frequency of HLA-DR^−^CD33^+^CD11b^+^ MDSCs and CD14^+^ Mo-MDSCs, the Ann Arbor Stage, LDH level, KPI and IPI scores were significant prognostic indicators for survival (*P* < 0.05). After adjusting for the key clinical prognostic factors and using a multivariate Cox regression analysis (Table 1), the HLA-DR^−^CD33^+^CD11b^+^ MDSCs and CD14^+^ Mo-MDSCs remained significant and independent predictors of DFS (*P* = 0.013, HR 21.633, 95 % CI 1.892–247.378; *P* = 0.016, HR 7.873, 95 % CI 1.467–42.238) and OS (*P* = 0.017, HR 19.593, 95 % CI 1.694–226.646; *P* = 0.027, HR 6.867, 95 % CI 1.243–37.948) in ENKL patients.

**Table 1:** Univariate and multivariate Cox regression analysis for DFS and OS of 32 patients with ENKL

Variables	Univariate analysis		Multivariate analysis	
	HR (95 % CI)	*P* value	HR (95 % CI)	*P* value
In MDSC population				
Disease-free survival				
Age (<40/≥40)	1.259 (0.355–4.470)	0.722		
Gender (female/male)	1.930 (0.544–6.852)	0.309		
Ann Arbor stage (I/II–IV)	3.434 (0.959–12.293)	0.045*	1.748 (0.368–8.295)	0.482
Subtypes (UNKTL/EUNKTL)	1.306 (0.277–6.164)	0.736		
B symptoms (no/yes)	55.563 (0.323–9550.869)	0.126		
LDH level (normal/elevated)	3.834 (1.091–13.474)	0.036*	0.875 (0.070–10.912)	0.918
KPI score (0–1/2–4)	3.417 (0.881–13.250)	0.076		
PIT score (0–1/2–4)	3.031 (0.852–10.785)	0.084		
IPI score (0–1/2–5)	3.718 (1.074–12.874)	0.038*	5.327 (0.324–87.663)	0.242
MDSC frequency (low/high)^a^	10.216 (1.285–81.244)	0.028*	21.633 (1.892–247.378)	0.013*
Overall survival				
Age (<40/≥40)	1.370 (0.342–5.491)	0.656		
Gender (female/male)	1.798 (0.449–7.198)	0.407		
Ann Arbor stage (I/II–IV)	3.894 (0.969–15.650)	0.046*	2.090 (0.374–11.678)	0.401
Subtypes (UNKTL/EUNKTL)	1.515 (0.315–7.320)	0.603		
B symptoms (no/yes)	53.653 (0.233–12,381.115)	0.151		
LDH level (normal/elevated)	5.011 (1.318–19.059)	0.018*	1.155 (0.085–15.622)	0.914
KPI score (0–1/2–4)	2.723 (0.680–10.908)	0.157		
PIT score (0–1/2–4)	4.063 (1.014–16.290)	0.048*	1.284 (0.095–17.339)	0.851
IPI score (0–1/2–5)	4.814 (1.289–17.977)	0.019*	6.153 (0.361–104.775)	0.209
MDSC frequency (low/high)	8.644 (1.073–69.636)	0.043*	19.593 (1.694–226.646)	0.017*
In Mo-MDSC population				
Disease-free survival				
Age (<40/≥40)	1.259 (0.355–4.470)	0.722		
Gender (female/male)	1.930 (0.544–6.852)	0.309		
Ann Arbor stage (I/II–IV)	3.434 (0.959–12.293)	0.045*	1.549 (0.402–5.970)	0.525
Subtypes (UNKTL/EUNKTL)	1.306 (0.277–6.164)	0.736		
B symptoms (no/yes)	55.563 (0.323–9550.869)	0.126		
LDH level (normal/elevated)	3.834 (1.091–13.474)	0.036*	1.843 (0.211–16.130)	0.581
KPI score (0–1/2–4)	3.417 (0.881–13.250)	0.076		
PIT score (0–1/2–4)	3.031 (0.852–10.785)	0.084		
IPI score (0–1/2–5)	3.718 (1.074–12.874)	0.038*	3.181 (0.358–28.269)	0.299
Mo-MDSC frequency (low/high)^a^	5.956 (1.249–28.411)	0.025*	7.873 (1.467–42.238)	0.016*
Overall survival				
Age (<40/≥40)	1.370 (0.342–5.491)	0.656		
Gender (female/male)	1.798 (0.449–7.198)	0.407		
Ann Arbor stage (I/II–IV)	3.894 (0.969–15.650)	0.046*	2.275 (0.436–11.878)	0.330
Subtypes (UNKTL/EUNKTL)	1.515 (0.315–7.320)	0.603		
B symptoms (no/yes)	53.653 (0.233–12,381.115)	0.151		
LDH level (normal/elevated)	5.011 (1.318–19.059)	0.018*	2.684 (0.240–30.257)	0.423
KPI score (0–1/2–4)	2.723 (0.680–10.908)	0.157		
PIT score (0–1/2–4)	4.063 (1.014–16.290)	0.048*	0.544 (0.039–7.604)	0.651
IPI score (0–1/2–5)	4.814 (1.289–17.977)	0.019*	4.432 (0.342–57.364)	0.254
Mo-MDSC frequency (low/high)	4.890 (1.004–23.809)	0.049*	6.867 (1.243–37.948)	0.027*

*DFS* disease-free survival, *OS* overall survival, *HR* hazard ratio, *CI* confidence interval, *LDH* lactate dehydrogenase, *IPI* International Prognostic Index, *KPI* Korean Prognostic Index, *PIT* Peripheral T cell lymphoma Prognostic Index

* Significant difference

^a^MDSC (high/low) is based on the median value of the MDSC density

## Discussion

It has been suggested that tumor pathogenesis is linked to immune imbalance and immune cell dysfunction. In this regard, tumors are found to affect myelopoiesis and induce the expansion of myeloid cells with immunosuppressive activity in tumor-bearing hosts, including animal models and human patients [18–22]. In this study, we found an expansion of HLA-DR^−^CD33^+^CD11b^+^ and HLA-DR^−^CD33^−^CD11b^+^ cells in the peripheral blood of ENKL patients. However, only the density of HLA-DR^−^CD33^+^CD11b^+^ MDSCs and not that of HLA-DR^−^CD33^−^CD11b^+^ cells was a significant and independent predictor for ENKL patient survival. This result was in line with our study on nasopharyngeal carcinoma (NPC) (unpublished data) and indicated that CD33 expression is an important marker for the MDSC population in cancer patients. Although the HLA-DR^−^CD33^−^CD11b^+^ cell population was expanded in ENKL patients, no clinical relevance and prognostic value was found in this cell population, and this cell population lacked the phenotypic features of MDSCs (Supplementary Figure 1). Our observations indicated that the immune-suppressive cell subset of HLA-DR^−^CD33^+^CD11b^+^ MDSCs has a prognostic value similar to that of Treg cells and other clinical parameters, including TNM stage, IPI score, and LDH level, in ENKL [23, 24].

Human MDSCs constitute a heterogeneous group. The definitive identification of human MDSCs is complicated by a lack of a specific marker and by the absence of a human homolog of mouse Gr-1 [12, 25, 26]. Human MDSCs include the Mo-MDSC and the PMN-MDSC subsets and, according to recent data, the myeloid subset, which has suppressive activity. The MDSC phenotypes are commonly evaluated using a single multicolor staining protocol for MDSC1–MDSC6 as follows: MDSC1 (CD14^+^IL-4Rα^+^); MDSC2 (CD15^+^ IL-4Rα^+^); MDSC3 (Lineage^−^ HLA-DR^−^ CD33^+^); MDSC4 (CD14^+^HLA-DR^low/−^); MDSC5 (CD11b^+^CD14^−^CD15^+^); and MDSC6 (CD15^+^ FSC^low^ SSC^high^) [27]. The MDSC phenotype varies by differentiation status and function in response to the environmental conditions of different cancers, and the MDSC phenotype has been defined as the HLA-DR^−^CD33^+^CD11b^+^ cell population, including PMN- and Mo-MDSCs, in many human cancers, including multiple myeloma [12, 27]. Based on our observations and those of others, ENKL-MDSCs were immunophenotyped as an HLA-DR^−^CD33^+^CD11b^+^ cell population in this study.

The ENKL-MDSC population consisted predominantly of CD14^+^ Mo-MDSCs with a minority of CD15^+^ PMN-MDSCs. Compared to healthy controls, however, the proportion of Mo-MDSCs in ENKL-MDSCs was decreased, and the proportion of PMN-MDSCs in ENKL-MDSCs was increased. The ENKL-MDSC population highly expressed immune mediator molecules, including Arg-1 and iNOS, and it expressed a low level of CD66b. Furthermore, these ENKL-MDSCs secreted moderate levels of suppressive cytokines, including IL-17, IL-10 and TGFβ, and they did not secrete the IFNγ inflammatory cytokine (data not shown). Compared with MDSCs from healthy donors, the ENKL-MDSCs expressed significant higher level of Arg-1 and iNOS, and they secreted higher levels of IL-17 (*P* < 0.05).

MDSCs can suppress T cell activation and proliferation in tumor-bearing hosts [28]. Our previous study and other studies have identified that human MDSCs from solid tumors or multiple myeloma can suppress anti-CD3-induced autologous or allogeneic T cell proliferation, including CD4^+^ and CD8^+^ T cells. There have been reports indicating that MDSC suppression requires antigen presentation through major histocompatibility complex (MHC) class I molecules [25, 29–33]. However, some studies have suggested that the MDSC suppression is dependent on innate immune sensing and that the MDSC-mediated T cell inhibition is a result of the activation of iNOS, leading to increased production of NO and ROS. Thus, the activated antigen-specific CD4^+^ T cells interact with MDSCs loaded with specific antigens, converting these cells to non-specific suppressors in cancers [16, 34]. In this study, we observed that ENKL-MDSCs strongly suppressed the OKT3-stimulated allogeneic or autologous CD4 T cell proliferation but that they only slightly suppressed the OKT3-stimulated allogeneic and autologous CD8 T cell proliferation. These results indicated that the suppression of T cell proliferation by ENKL-MDSCs is both antigen specific and non-antigen specific, especially for CD4 T cell proliferation. Furthermore, our data were in line with the suggestion that MDSCs from tumor-bearing hosts, as characterized by a high level of iNOS/NOS2 and Arg-1, are potent inhibitors of Ag-specific T cell functions that are able to suppress T cells in an Ag-independent manner [5, 13, 20, 35–40]. Furthermore, our results showed that blockage of iNOS, Arg-1 and ROS recovered the MDSC-mediated inhibition of anti-CD3-induced allogeneic and autologous PBMC proliferation. Interestingly, our observations suggested that the inhibition of T cell proliferation by ENKL-MDSCs also correlated with suppressed cytokine secretion, including IL-10 and TGFβ, as well as induction of Treg cells, which was in line with other reports in solid cancers [41, 42]. Our observations suggested that multiplex mechanisms that include NO production, ROS production, cytokine induction (IL-10 and TGFβ), and Treg cell induction are involved in ENKL-MDSC-mediated suppression.

The percentage or the frequency of MDSC population is always correlated with poor survivals of cancer patients [43, 44]. Here, our study demonstrated that the HLA-DR^−^CD33^+^CD11b^+^ MDSC population was an independent poor prognostic indicator for DFS and OS of ENKL patients. Our data further showed that the Mo-MDSC population, but not the PMN-MDSC population, is an independent predictor for DSF and OS in ENKL patients. These observations may explain why the Mo-MDSCs were the main component of the MDSC population in ENKL patients. Our results are in line with reports by others. Some studies have indicated that the CD14^+^ MDSC population is associated with disease progression in cancers [29, 45, 46].

In addition, the number of circulating IL-17-producing MDSCs correlated with patient DFS and OS. IL-17 is an inflammatory cytokine typically secreted by CD4 Th17 and CD8 Tc17 cells [47]. Recent findings have indicated that the role of IL-17 in tumor development is controversial, and IL-17 could promote the induction of MDSCs at a tumor site and enhance the suppressive function of MDSCs on T cell proliferation [48–52]. Our observations for the first time indicate that ENKL-MDSCs can secrete higher levels of IL-17 compared to healthy donors and that the number of IL-17-producing MDSCs is correlated with ENKL patient prognosis (Supplementary Figure 2). A functional investigation of IL-17-producing MDSCs should be performed in future studies.

## Conclusions

This study analyzed for the first time the phenotypic and functional properties as well as the clinical significance of the MDSC population in ENKL patients. Our results revealed an expansion of circulating MDSCs in ENKL patients; these ENKL-MDSCs mainly consist of CD14^+^ Mo-MDSCs and express high levels of iNOS, Arg-1 and suppressive cytokines including IL-10, TGFβ and IL-17. The ENKL-MDSC-mediated suppression of OKT3-stimulated allogeneic and autologous T cell proliferation is dependent on iNOS, Arg-1 and ROS activities and is correlated with cytokine changes and Treg cell induction. Moreover, increases in circulating MDSCs and Mo-MDSCs correlate with poor DFS and OS in patients, and they are independent predictors in ENKL. Collectively, these findings demonstrate a novel role and mechanism of MDSCs in the tumor pathogenesis of ENKL, thus unveiling a new avenue for ENKL immunotherapy.

### Electronic supplementary material

Below is the link to the electronic supplementary material.

Supplementary material 1 (PDF 457 kb)

## Abbreviations

CFSE: Carboxyfluorescein diacetate succinimidyl ester
DFS: Disease-free survival
EBNA1: EBV nuclear antigen 1
EBV: Epstein–Barr virus
ENKL: Extranodal natural killer (NK)/T cell lymphoma
HD: Healthy donor
IFNγ: Interferon gamma
iNOS: Inducible nitric oxide synthase
IPI: International Prognostic Index
KPI: Korean Prognostic Index
LDH: Lactate dehydrogenase
LMP1: Latent membrane protein-1
LMP2: Latent membrane protein-2
l-NMMA: NG-methyl l-arginine
LPS: Lipopolysaccharide
MDSCs: Myeloid-derived suppressor cells
MHC: Major histocompatibility complex
Mo-MDSCs: Monocytic MDSCs
NAC: *N*-acetylcysteine
NO: Nitric oxide
NOHA: *N*-hydroxy-nor-l-arginine
NPC: Nasopharyngeal carcinoma
OS: Overall survival
PBMCs: Peripheral blood mononuclear cells
PMN-MDSCs: Polymorphonuclear MDSCs
ROS: Reactive oxygen species
SEM: Standard error of mean
TGFβ: Transforming growth factor beta
UNKTL: Upper aerodigestive tract NK/T cell lymphoma
