# Supplementary material for: Myeloid-derived suppressor cells inhibit T cell proliferation in human extranodal NK/T cell lymphoma: a novel prognostic indicator
Source: Cancer Immunol Immunother. 2015 Oct 23;64(12):1587–99. doi: 10.1007/s00262-015-1765-6 (PMC4643115; doi:10.1007/s00262-015-1765-6)
Supplement: Supplementary file 1 — Supplementary material 1 (PDF 457 kb) [file 262_2015_1765_MOESM1_ESM.pdf]

Supplementary file

Supplementary Figure 1

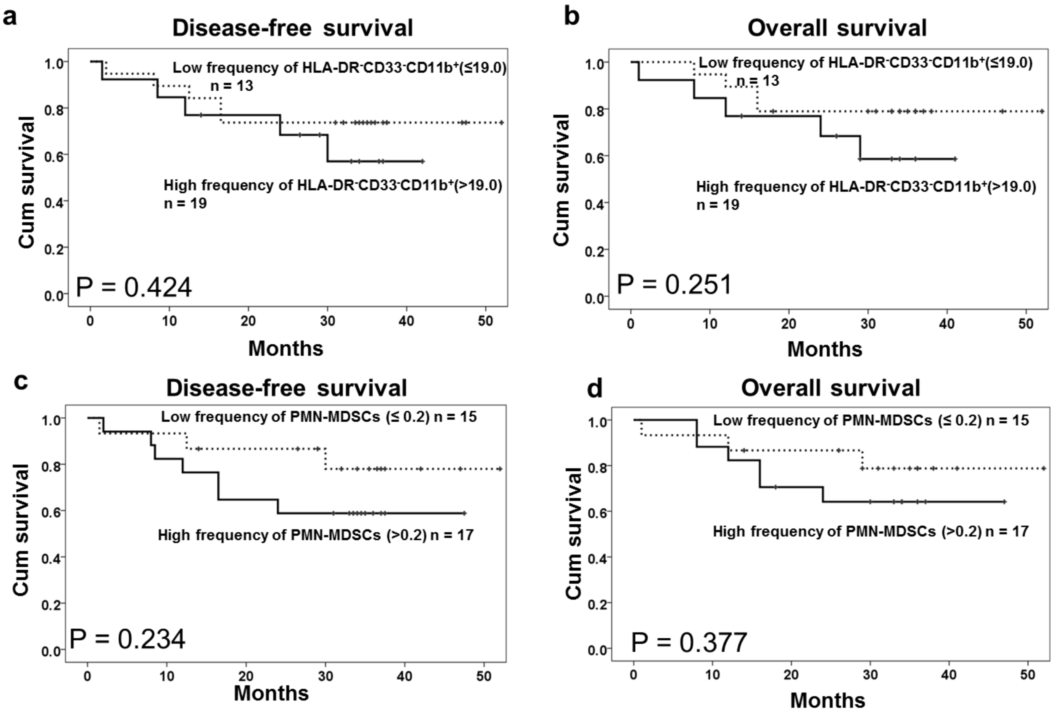

Supplementary Figure 1 The correlation of circulating HLA-DR$^-$CD33$^-$CD11b$^+$ cells and CD15$^+$ PMN-MDSCs with the survival of ENKL patients. (a-b) The DFS and OS curves for the patients according to the low and high numbers of HLA-DR$^-$CD33$^-$CD11b$^+$ cells ($P > 0.05$, log-rank test). (c-d) The DFS and OS curves for the patients according to the low and high numbers of CD15+ PMN-MDSCs ($P > 0.05$, log-rank test).

Supplementary Figure 2

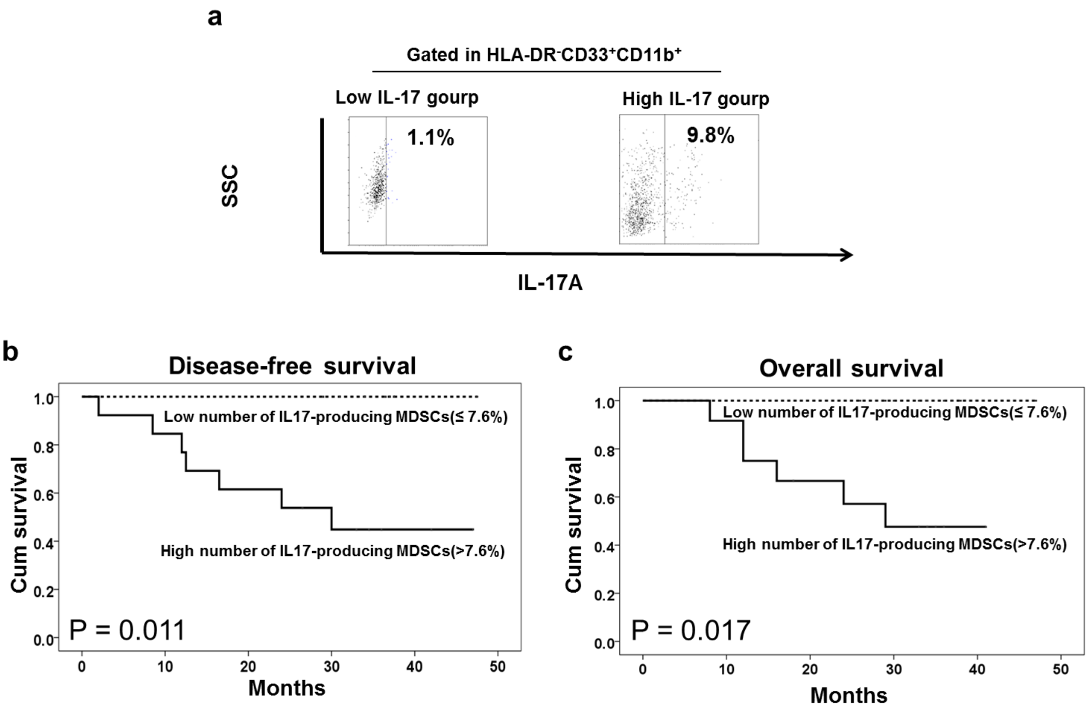

Supplementary Figure 2 The correlation of circulating IL17-producing MDSCs with DFS and OS in extranodal NK/T-cell lymphoma cases. (a) The dot plots represent the low and high numbers of IL17-producing MDSCs gating on the MDSC fraction among the PBMCs from four patients with ENKL. (b-c) The DFS and OS curves for the patients according to the low and high numbers of IL17-producing MDSCs (P = 0.011 and 0.017, respectively; log-rank test). The cut-off value was the median of the IL17-producing MDSC density.

Supplementary Table 1. Clinical characteristics of 32 patients with ENKL

Clinicopathological parameters	Cases (%)
**No. of cases**	32
**Age (y)**	
Mean	40.5
Range	17-70
**Gender**	
Female	9 (28.1)
Male	23 (71.9)
**Ann Arbor Stage**	
I	19 (59.4)
II	3 (9.4)
III	3 (9.4)
IV	7 (21.9)
**Subtypes**	
UNKTL	27 (84.4)
EUNKTL	5 (15.6)
**B symptoms**	
No	12 (37.5)
Yes	20 (62.5)
**LDH level**	
Normal	23 (71.9)
Elevated	9 (28.1)
**KPI score**	
0-1	17 (53.1)
2-4	15 (46.9)
**PIT score**	
0-1	20 (62.5)
2-4	12 (37.5)
**IPI score**	
0-1	24 (75.0)
2-5	8 (25.0)
**Death**	
No	23 (71.9)
Yes	9 (28.1)

LDH, lactate dehydrogenase; IPI, International Prognostic Index; KPI, Korean Prognostic Index; PIT, Prognostic Index for Peripheral T cell lymphoma.

Supplementary Table 2. Antibody information

Name	Lot number	Clone number	Brand	Producing area
CD14-FITC	11-0149-42	61D3	eBioscience	San Diego, CA, USA
CD15-FITC	11-0159-41	H19B	eBioscience	San Diego, CA, USA
HLA-DR-FITC	11-9952-42	L243	eBioscience	San Diego, CA, USA
HLA-DR-PE	555812	/	BD Bioscience	San Jose, CA, USA
CD66b-APC	17-0666-41	G10F5	eBioscience	San Diego, CA, USA
CD11b-PE-Cy7	25-0118-42	ICRF44	eBioscience	San Diego, CA, USA
IL17a-PE	12-7179-42	eBio64DEC17	eBioscience	San Diego, CA, USA
IFN-γ-APC	17-7319-82	4S.B3	eBioscience	San Diego, CA, USA
IL-10-PE	559330	/	BD Bioscience	San Jose, CA, USA
TGF-β1（LAP）	349606	TW4-2F8	BioLegend	San Diego, CA, USA
CD33-PerCP/Cy5.5	303414	WM53	BioLegend	San Diego, CA, USA
NOS2-FITC	sc-7271	/	Santa Cruz	Dallas, TX, USA
Arg-1-FITC	IC5868F	/	Santa Cruz	Dallas, TX, USA

Supplementary Table 3. The association of the frequency of circulating MDSCs with the clinical parameters of 32 patients with ENKL

Clinicopathological parameters	Total cases	High level of HLA-DR$^-$CD33$^+$CD11b$^+$ (%)	*P* value	High level of HLA-DR$^-$CD33$^-$CD11b$^+$ (%)	*P* value
**Age**					
<40 y	15	10 (66.7)	0.265	10 (66.7)	0.430
≥40 y	17	8 (47.1)		9 (52.9)	
**Gender**					
Female	9	7 (77.8)	0.125	7 (77.8)	0.185
Male	23	11 (47.8)		12 (52.2)	
**Ann Arbor Stage**					
I	19	10 (52.6)	0.618	14 (73.7)	0.071
II-IV	13	8 (61.5)		5 (38.5)	
**Subtypes**					
UNKTL	27	15 (55.6)	0.854	16 (59.3)	0.975
EUNKTL	5	3 (60.0)		3 (60.0)	
**LDH level**					
Normal	23	12 (52.2)	0.457	13 (56.5)	0.599
Elevated	9	6 (66.7)		6 (66.7)	
**B symptoms**					
No	12	5 (41.7)	0.198	8 (66.7)	0.515
Yes	20	13 (65.0)		11 (55.0)	
**KPI score**					
0-1	17	10 (58.8)	0.755	11 (64.7)	0.513
2-4	15	8 (53.3)		8 (53.3)	
**PIT score**					
0-1	20	12 (60.0)	0.581	13 (65.0)	0.403
2-4	12	6 (50.0)		6 (50.0)	
**IPI score**					
0-1	24	14 (58.3)	0.681	14 (58.3)	0.835
2-5	8	4 (50.0)		5 (62.5)	

P value as determined by Pearson's χ^2 test.

Supplementary Table 4. The association of the frequency of circulating Mo-MDSCs and PMN-MDSCs with the clinical parameters of 32 patients with ENKL

**Clinicopathological parameters**	**Total cases**	**High level of Mo-MDSCs (%)**	***P* value**	**High level of PMN-MDSCs (%)**	***P* value**
**Age**					
<40 y	15	8 (53.3)	0.492	10 (66.7)	0.149
≥40 y	17	7 (41.2)		7 (41.2)	
**Gender**					
Female	9	7 (77.8)	**0.028***	4 (44.4)	0.538
Male	23	8 (34.8)		13 (56.5)	
**Ann Arbor Stage**					
I	19	8 (42.1)	0.513	12 (63.2)	0.169
II-IV	13	7 (53.8)		5 (38.5)	
**Subtypes**					
UNKTL	27	12 (44.4)	0.522	15 (55.6)	0.522
EUNKTL	5	3 (60.0)		2 (40.0)	
**LDH level**					
Normal	23	11 (47.8)	0.863	12 (52.2)	0.863
Elevated	9	3 (44.4)		5 (55.6)	
**B symptoms**					
No	12	5 (41.7)	0.647	5 (41.7)	0.314
Yes	20	10 (50.0)		12 (60.0)	
**KPI score**					
0-1	17	7 (41.2)	0.492	10 (58.8)	0.492
2-4	15	8 (53.3)		7 (46.7)	
**PIT score**					
0-1	20	9 (45.0)	0.784	12 (60.0)	0.314
2-4	12	6 (50.0)		5 (41.7)	
**IPI score**					
0-1	24	12 (50.0)	0.539	14 (58.3)	0.306
2-5	8	3 (37.5)		3 (37.5)	

P value as determined by Pearson's χ^2 test. *, $P < 0.05$.
